# Supplementary material for: Unravelling three-dimensional adsorption geometries of PbSe nanocrystal monolayers at a liquid-air interface
Source: Commun Chem. 2020 Mar 2;3:28. doi: 10.1038/s42004-020-0275-4 (PMC9814399; doi:10.1038/s42004-020-0275-4)
Supplement: Supplementary file 1 — Supplementary Information [file 42004_2020_275_MOESM1_ESM.pdf]

Supporting Information

Unravelling three-dimensional adsorption geometries of PbSe nanocrystal monolayers at a liquid-air interface

Jaco J. Geuchies[1,2,*], Giuseppe Soligno[1], Ellenor Geraffy[1], Cedric P. Hendrikx[2], Carlo van Overbeek[1], Federico Montanarella[1], Marlou R. Slot[1], Oleg V. Konovalov[2], Andrei V. Petukhov[‖,3] and Daniel Vanmaekelbergh[1]

1 Condensed Matter and Interfaces, & Physical and Colloid Chemistry, Debye Institute for Nanomaterials Science, Utrecht University, P.O. Box 80000, 3508 TA Utrecht, The Netherlands.

2 ESRF – The European Synchrotron, ID10, 71 Rue des Martyrs, 38000 Grenoble, France.

3 Laboratory of Physical Chemistry, Department of Chemical Engineering and Chemistry, Eindhoven University of Technology, P.O. Box 513, 5600 MB, Eindhoven, Netherlands.

* Present address: Optoelectronic Materials Section, Faculty of Applied Sciences, Delft University of Technology, Van der Maasweg 9, 2629 HZ Delft, The Netherlands.

SUPPLEMENTARY METHODS

Nanocrystal synthesis.

The PbSe nanocrystals used for the oriented attachment experiments in this study were prepared using the method described in ref. 1[1]. The synthesis was performed in a glovebox with a water- and oxygen-free environment. (a) 4.77 g of lead acetate trihydrate (99.999% Aldrich), 10.35 g of oleic acid (OA, 90% Aldrich) and 39.75 g octadecene (ODE, 90% Aldrich) were heated to 130 °C under low pressure (10^{-3} bar) for approximately 4 h. (b) A second mixture containing 3.52 g Se (99.999% Alfa Aesar), 46.59 ml trioctylphosphine (90% Fluka) and 0.41 ml diphenylphosphine (98% Aldrich) was prepared by dissolving the Se. Subsequently, solution (a) was heated in a three-necked round-bottom flask to 180 °C after which 15 ml of solution (b) was rapidly injected. The particles were grown for approximately 60 s, after which the reaction was quenched with 20 ml butanol. After the solution was cooled down to approximately 50 °C, 10 ml methanol was added to induce precipitation of the nanocrystals. The resulting suspension was centrifuged at approximately 1,200*g* for 10 min, the supernatant was removed and the washed particles were redispersed in toluene. This washing procedure was repeated two times.

To change the NC size, we varied the growth time, and added additional oleic acid to the Pb-oleate/ODE (which reduces the activity of the Pb precursor) mixture prior to injection of the Se precursor. The added amount of oleic acid, growth time and obtained NC diameter are summarized inSupplementary Table 1. TEM micrographs, results from image analysis and steady-state absorption spectra are shown in Figures S1-S3.

ESRF ID10 GISAXS/GIWAXS/XRR.

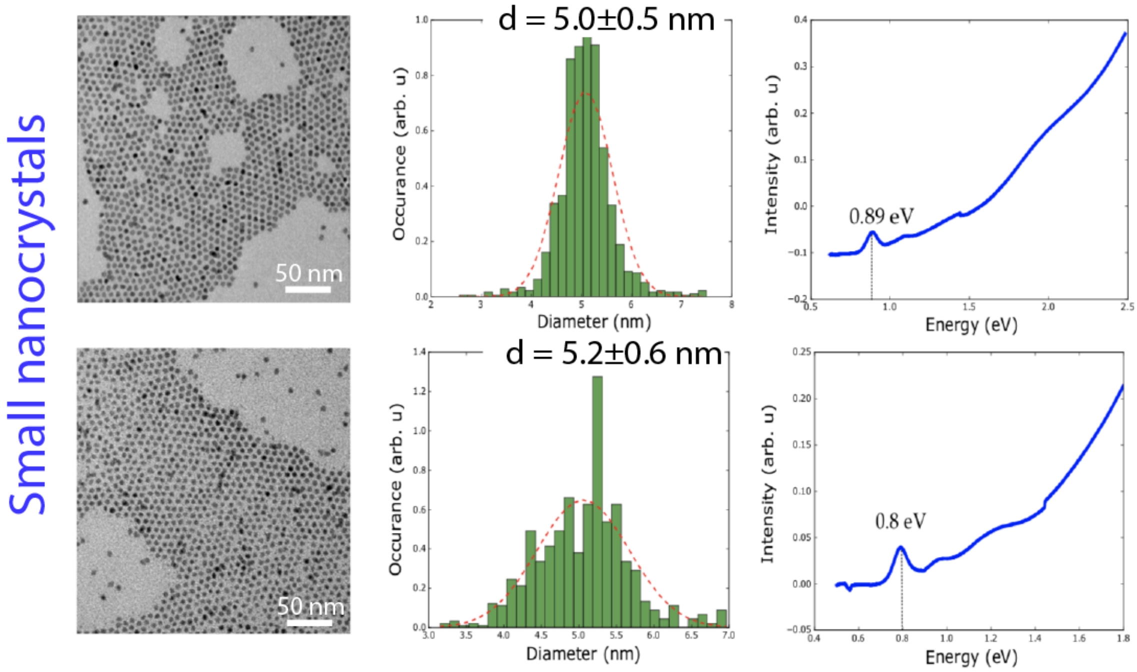

Supplementary Figure 1: TEM micrographs, results from image analysis and steady-state absorption spectra of the small PbSe NCs.

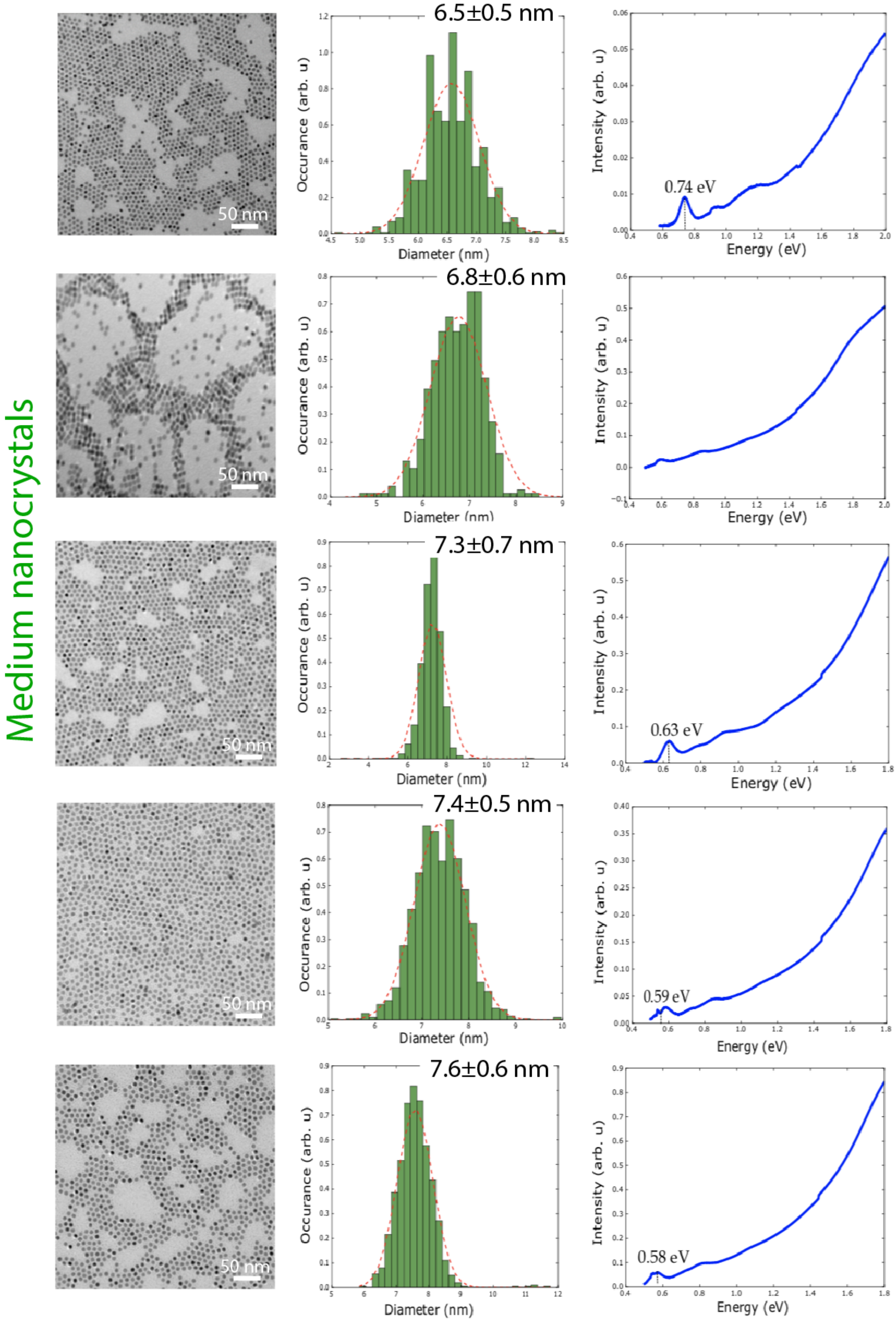

Supplementary Figure 2: TEM micrographs, results from image analysis and steady-state absorption spectra of the medium-sized PbSe NCs.

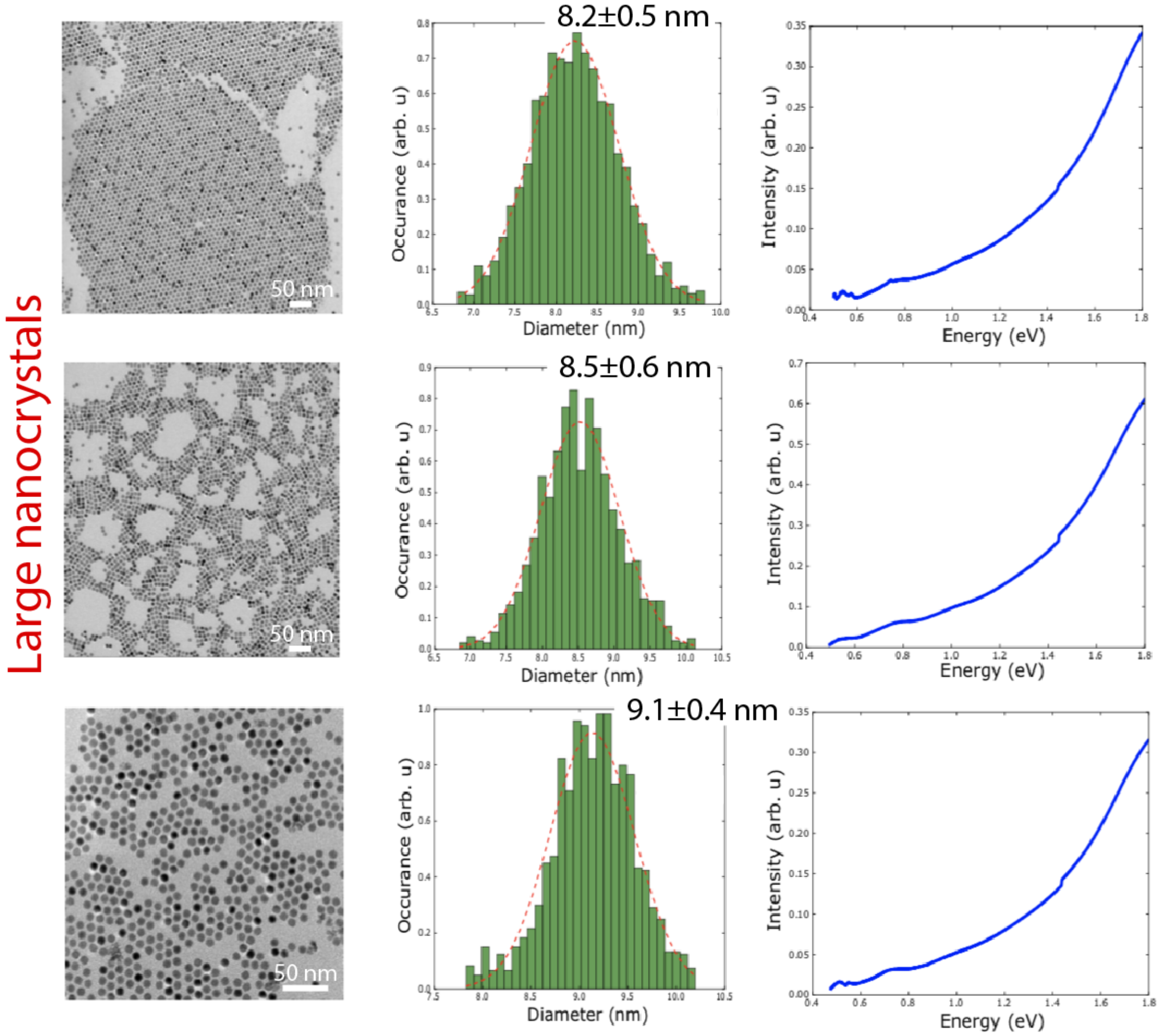

Supplementary Figure 3: TEM micrographs, results from image analysis and steady-state absorption spectra of the large PbSe NCs.

NC diameter ± σ (nm) - TEM	Added amount of oleic acid (mL)	Growth time (s)
5.0 ± 0.5	0	60
5.2 ± 0.6	0	90
6.5 ± 0.5	0.4	120
6.8 ± 0.6	0.4	140
7.3 ± 0.7	0.6	120
7.4 ± 0.5	0.6	150
7.6 ± 0.6	0.8	165
8.2 ± 0.5	0.8	200
8.5 ± 0.6	1	250
9.1 ± 0.4	1	360

Supplementary Table 1: NC diameter and growth parameters during synthesis.

NC diameter ± σ (nm) - TEM	Inter-NC distance ± σ (nm) - GISAXS
5.0 ± 0.5	6.1 ± 0.3
5.2 ± 0.6	6.53 ± 0.04
6.5 ± 0.5	8.3 ± 0.2
6.8 ± 0.6	8.8 ± 0.1
7.3 ± 0.7	8.2 ± 0.2
7.4 ± 0.5	8.6 ± 0.3
7.6 ± 0.6	8.7 ± 0.2
8.2 ± 0.5	8.7 ± 0.1
8.5 ± 0.6	8.5 ± 0.2
9.1 ± 0.4	8.6 ± 0.1

Supplementary Table 2: NC diameters as obtained by image analysis on TEM images, compared to the inter-NC distance as obtained from the in-situ GISAXS experiments, divided into the small (blue), medium (green) and large (red) diameter categories. NC-NC distance determination for small (hexagonal 2d lattice $4\pi/\text{sqrt}(3)q$) and large (distorted square $2\pi/q$).

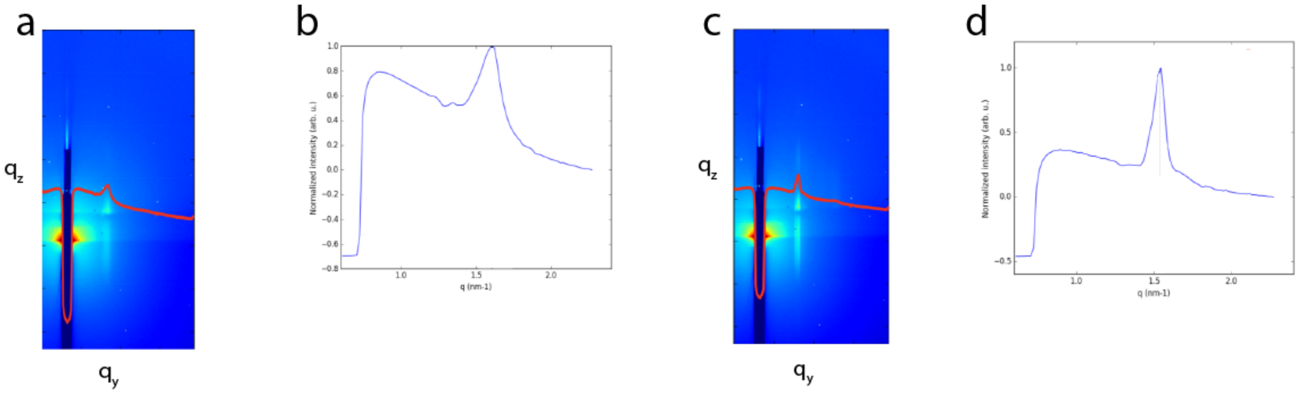

Supplementary Figure 4: GISAXS patterns of the NC-monolayers of the small NCs. (a) GISAXS pattern of the 6.5±0.5 nm diameter NCs, including intensity trace in the horizontal direction (b). (c) GISAXS pattern of the 5.2±0.6 nm diameter NCs, including intensity trace in the horizontal direction (d).

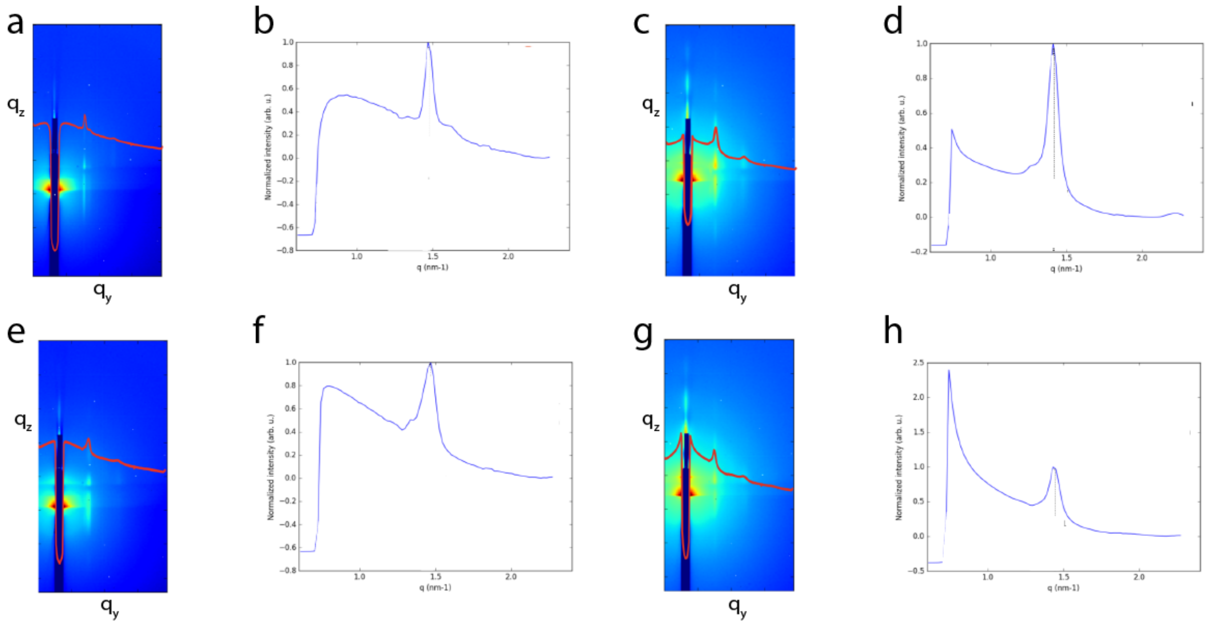

Supplementary Figure 5: GISAXS patterns of the NC-monolayers of the medium-sized NCs. (a) GISAXS pattern of the 5.0±0.5 nm diameter NCs, including intensity trace in the horizontal direction (b). (c) GISAXS pattern of the 6.8±0.6 nm diameter NCs, including intensity trace in the horizontal direction (d). (e) GISAXS pattern of the 7.3±0.7 nm diameter NCs, including intensity trace in the horizontal direction (f). (g) GISAXS pattern of the 7.4±0.5 nm diameter NCs, including intensity trace in the horizontal direction (h).

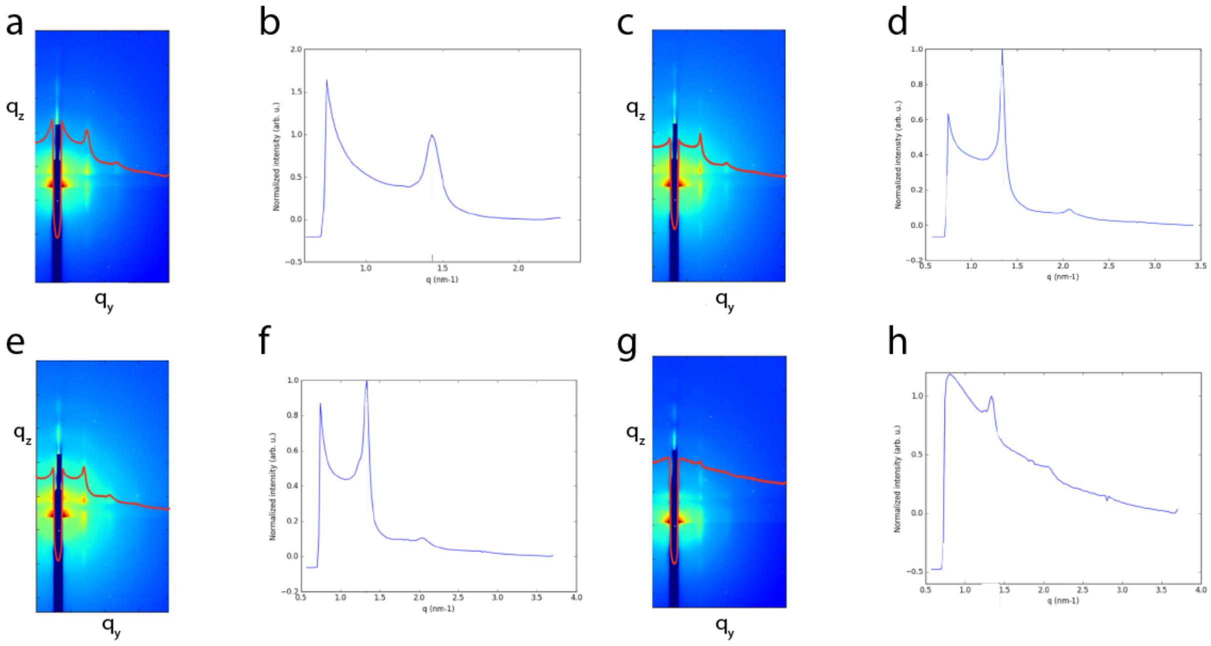

Supplementary Figure 6: GISAXS patterns of the NC-monolayers of the large NCs. (a) GISAXS pattern of the 7.6±0.6 nm diameter NCs, including intensity trace in the horizontal direction (b). (c) GISAXS pattern of the 8.2±0.5 nm diameter NCs, including intensity trace in the horizontal direction (d). (e) GISAXS pattern of the 8.5±0.6 nm diameter NCs, including intensity trace in the horizontal direction (f). (g) GISAXS pattern of the 9.1±0.4 nm diameter NCs, including intensity trace in the horizontal direction (h).

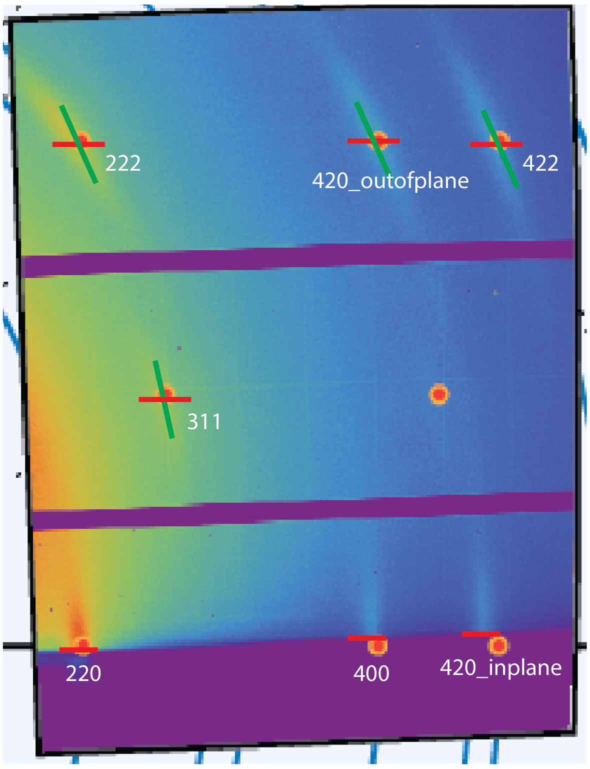

Supplementary Figure 7: Additional GIWAXS pattern, with all the atomic reflections labeled.

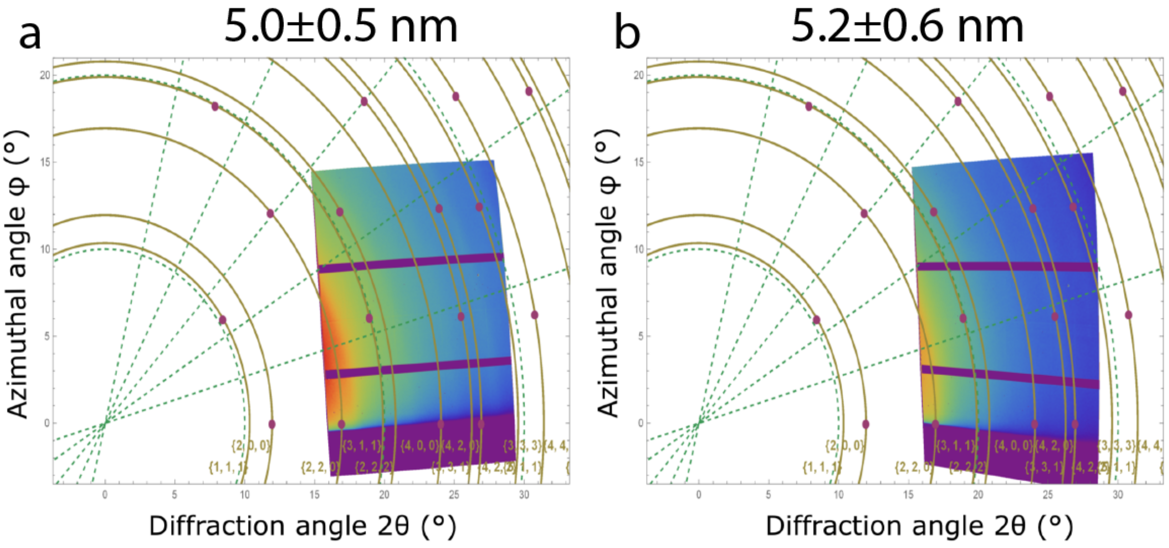

Supplementary Figure 8: GIWAXS pattern for the small nanocrystals. (a) GIWAXS pattern for the 5.0±0.5 nm nanocrystals. (b) GIWAXS pattern for the 5.2±0.6 nm nanocrystals. Notice that the scattered intensity is very small. The intensity scales with the V; smaller particles diffract less.

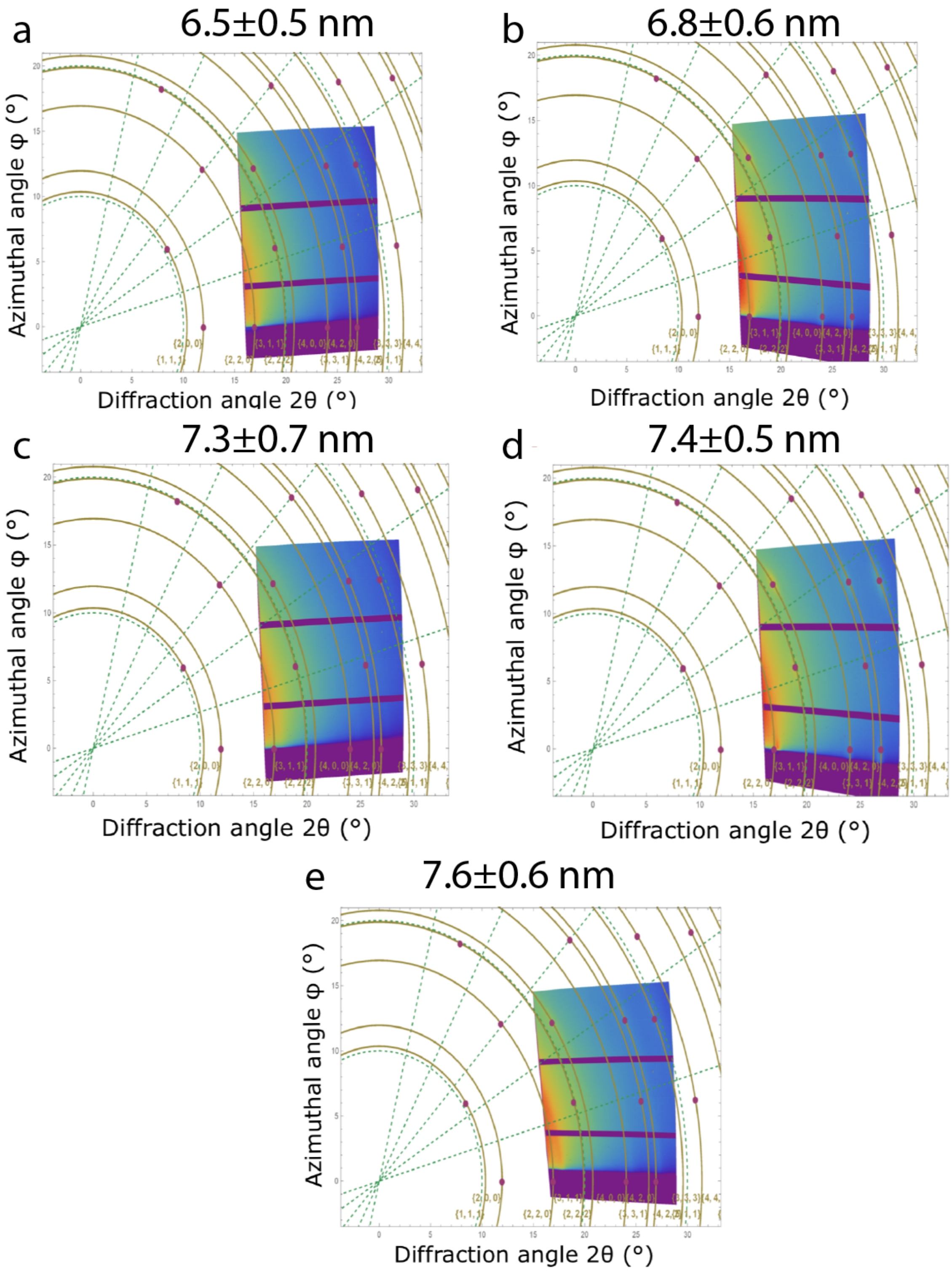

Supplementary Figure 9: GIWAXS pattern for the medium-sized nanocrystals. (a) GIWAXS pattern for the 6.5±0.5 nm nanocrystals. (b) GIWAXS pattern for the 6.8±0.6 nm nanocrystals. (c) GIWAXS pattern for the 7.3±0.7 nm nanocrystals. (d) GIWAXS pattern for the 7.4±0.5 nm nanocrystals. (e) GIWAXS pattern for the 7.6±0.6 nm nanocrystals. All samples show crystallographic alignment, where the {100} PbSe atomic facet is pointing upwards w.r.t. the liquid interface.

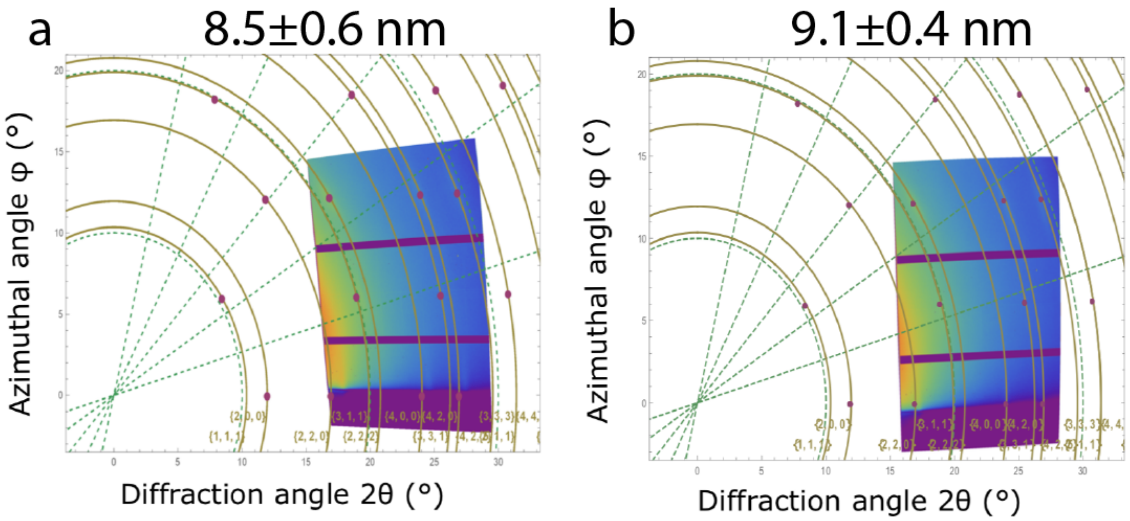

Supplementary Figure 10: GIWAXS pattern for the large nanocrystals. (a) GIWAXS pattern for the 8.5±0.5 nm nanocrystals. (b) GIWAXS pattern for the 9.1±0.4 nm nanocrystals.

NC diameter ± σ (nm) - TEM	222 FWHM (degrees)	422 FWHM (degrees)	420 FWHM (degrees)	311 FWHM (degrees)
6.5 ± 0.5	10	8	5	6
6.8 ± 0.6	7	6.5	6.5	6.5
7.4 ± 0.5	6	4.4	4.4	4
7.6 ± 0.6	6.5	5.5	4	6.5
8.2 ± 0.5	4	4	3.5	4.6
8.5 ± 0.6	4	4.5	3.5	5.5
9.1 ± 0.4	4.5	5	4.5	5.4

Supplementary Table 3: FWHM of the atomic reflections for the crystallographically aligned NCs from GIWAXS in the azimuthal (φ) direction. The small NCs did not show any crystallographic alignment, as discussed in the main text.

Specular X-ray Reflectivity (XRR) and analysis

The XRR data was fitted using a least-square fitting procedure employing a Parratt formalism. Here, we simulate a NC at the ethylene glycol-air interface, and stratify the density profile into N layers (by definition, the N'th layer is the bottom ethylene glycol layer). Each layer has a refractive index of the j'th layer, $n_j = 1 - \delta_j + i\beta_j$, and thickness Δj. The wavevector in each layer q_j is then calculated as

$$q_j = \sqrt{q^2 - 8k^2\delta_j + 8ik^2\beta_j} \qquad \text{(Supplementary equation 1)}$$

where k equals $2\pi/\lambda$. The reflectivity of the j'th layer can be calculated using the Fresnel relation

$$r_{j,j+1} = \frac{q_j - q_{j+1}}{q_j + q_{j+1}} \qquad \text{(Supplementary equation 2)}$$

The first step is to calculate the reflectivity from the bottom N'th interface. As the substrate is infinitely thick, there are no multiple reflections to consider. From there, it can be iterated upwards through the layers until the top is reached, where the reflected intensity of each layer is calculated as

$$r_{j,j+1} = \frac{r_{j-2,j-1} + r_{j-1,j}p_{j-1}^2}{1 + r_{j-2,j-1}r_{j-1,j}p_{j-1}^2} \qquad \text{(Supplementary equation 3)}$$

Here, $p^2_j = \mathrm{Exp}(i\Delta_j q_j)$ is the phase factor, accounting for the difference in phase between photons scattered between the j'th and j+1'th layer. From the bottom, the reflected intensity is iterated recursively until the total reflected intensity, $r_{0,1}$, at the interface between vacuum and the first layer is obtained.

Method 1: Model independent solutions

As a first trial, we made a model independent fit of the data, i.e. we match the electron density profile to the experimental data. This gives a good estimate of the higher electron density features, such as the NC layer on top of the liquid. Using this method, however, we cannot ascribe any other properties to the NC layer, such as the adsorption height w.r.t. the liquid interface, etc. Some examples of the model independent approach, and comparison with the model dependent solutions are shown below.

Particle Diameter (nm)	6.5	8.5	9.1	6.8	5.0	7.6	7.3	5.2	6.5
χ^2	0.0006	0.0074	0.0003	0.0071	0.0003	0.0047	0.0006	0.0008	0.0029
Fishers criterion	1	1	1	1	1	0	1	1	1
Log-test	0.55	0.85	0.20	1.58	0.13	0.40	0.13	0.18	0.57
Fishers criterion	1	1	1	0	1	0	1	1	0
Particle Diameter (nm)	6.5	5.2	7.3	8.2	7.4	7.3	5.0	5.0	5.2
χ^2	0.0023	0.0045	0.0001	0.0067	0.0035	0.0048	0.0005	0.0019	0.0105
Fishers criterion	1	1	0	1	1	1	1	1	1
Log-test	0.36	0.44	0.15	2.22	0.48	0.51	0.21	0.26	0.48
Fishers criterion	1	1	1	0	1	1	1	1	1

Supplementary Table 4: Fit results for the model independent approach.

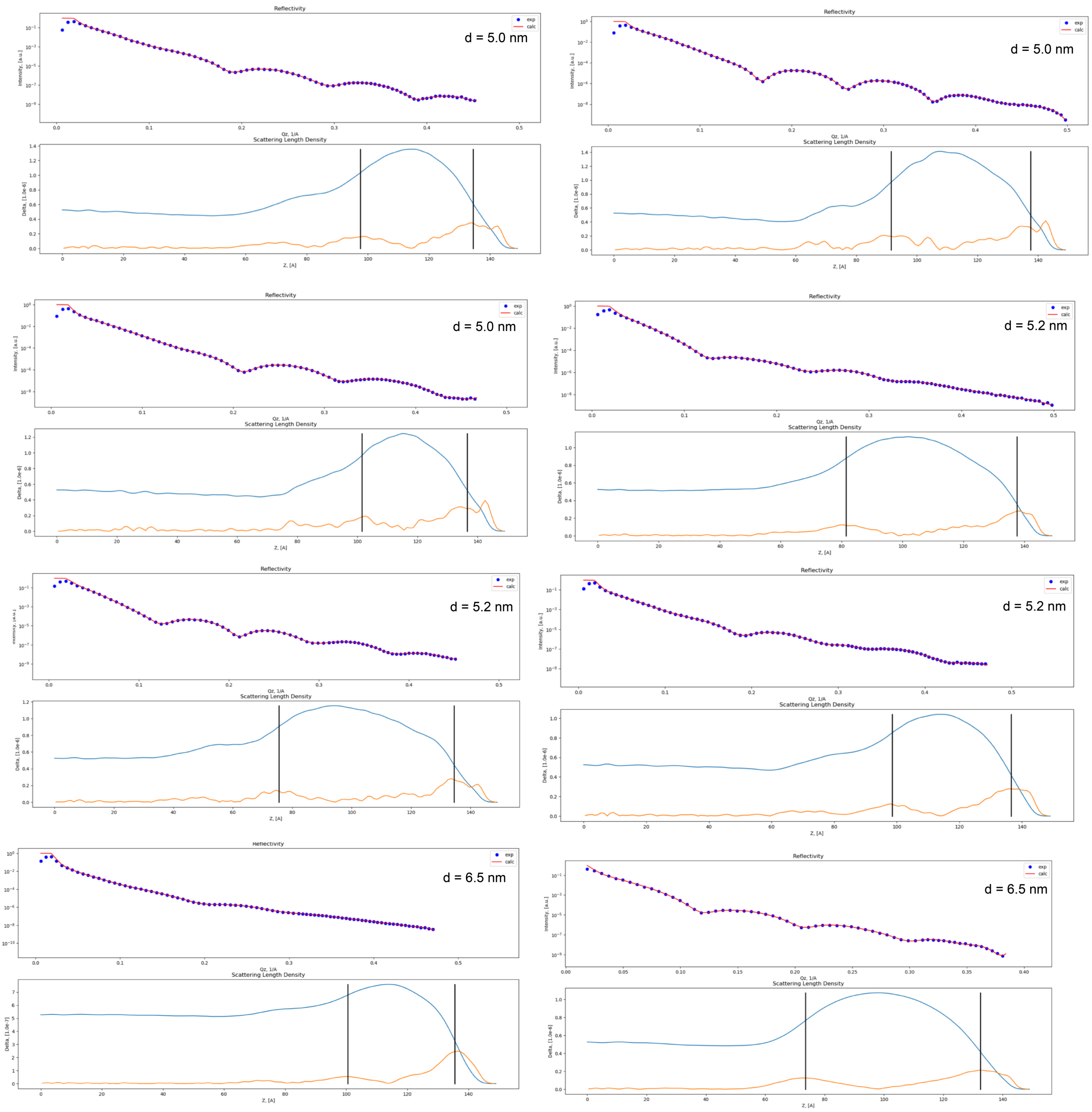

Supplementary Figure 11: XRR data with the model independent fit part 1. The above panels show the data and fit, the panels on the bottom show the density profile and first derivative of the density profile.

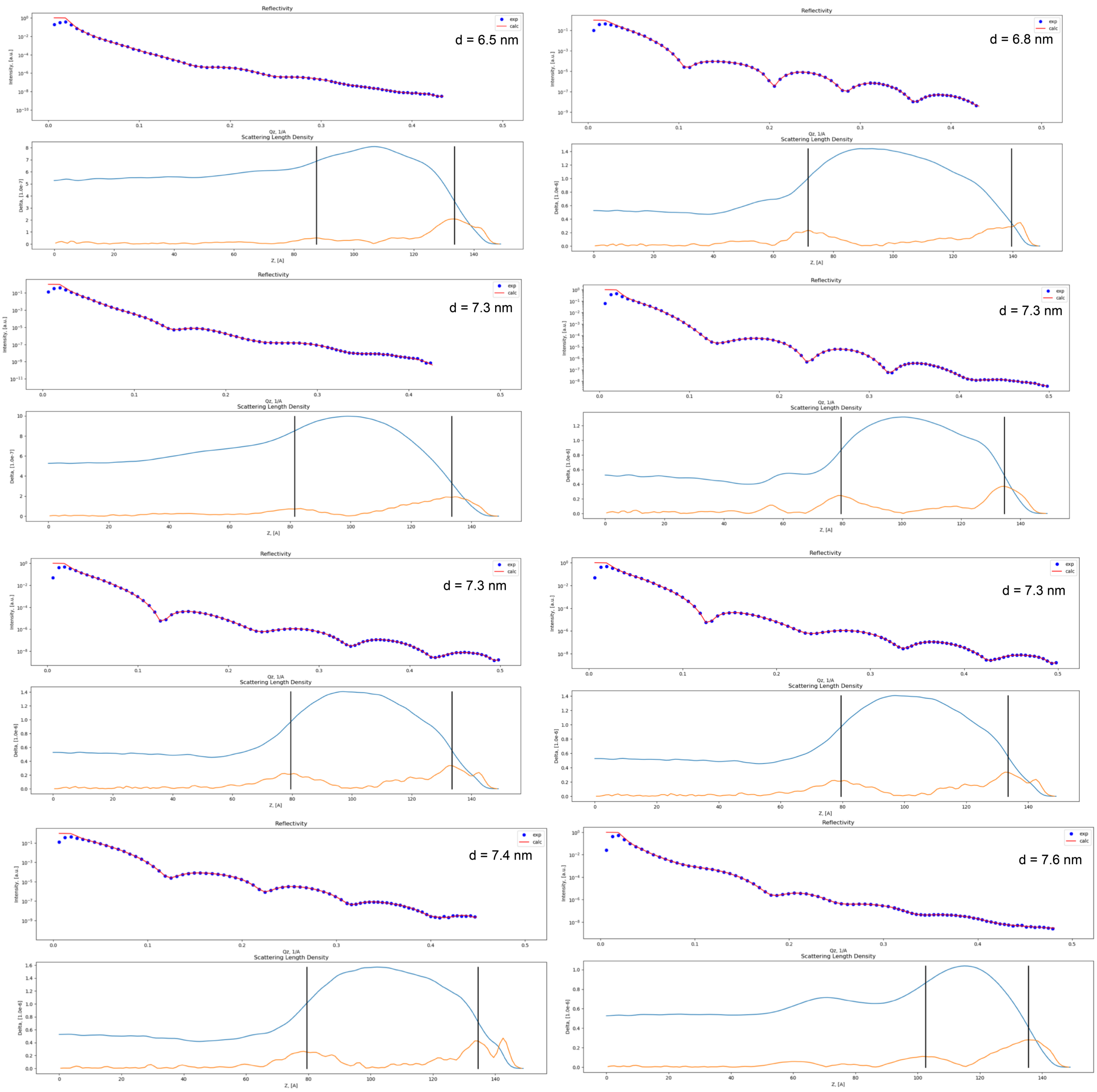

Supplementary Figure 12: XRR data with the model independent fit part 2. The above panels show the data and fit, the panels on the bottom show the density profile and first derivative of the density profile.

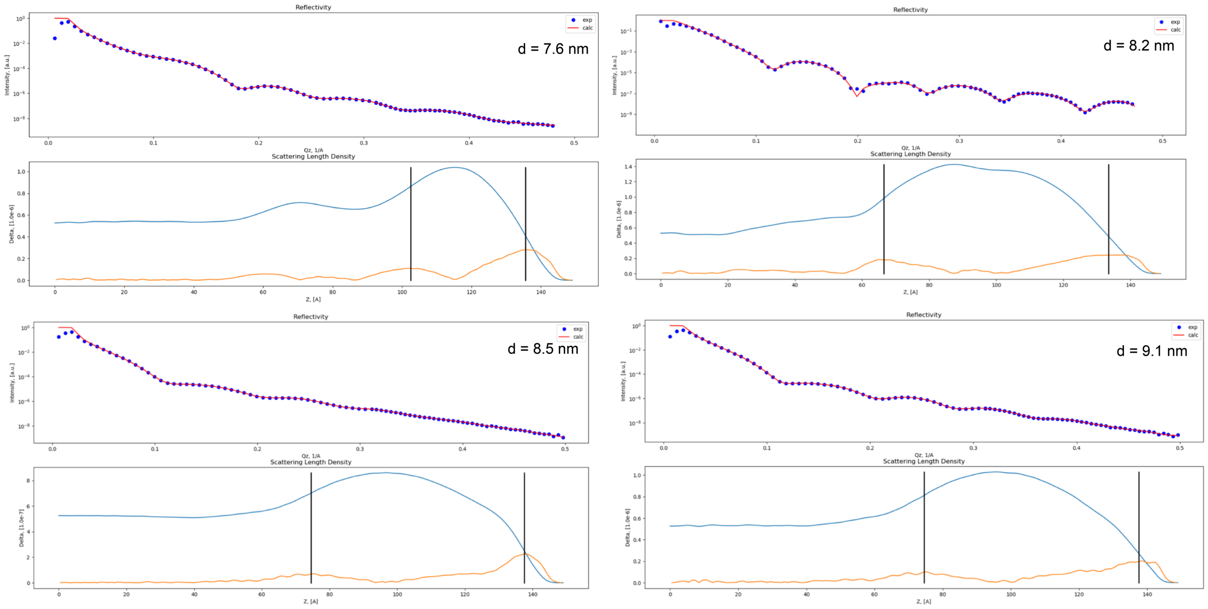

Supplementary Figure 13: XRR data with the model independent fit part 3. The above panels show the data and fit, the panels on the bottom show the density profile and first derivative of the density profiles.

Method 2: Modelling physically relevant properties and adsorption geometries

The parameters we tried to implement were the height of the NC center-of-mass with respect to the ethylene glycol interface, particle shape (degree of truncation and diameter), the ligand density and size of the ligand shell on the particles, Gaussian roughness of the ethylene glycol interface. We tried fitting the data both with a *single particle model*, i.e. there is only one adsorption height w.r.t. the interface, and a *double particle model*, where the center of mass of two of these particles can be displaced w.r.t. each other in the direction perpendicular to the interface (i.e. this creates a double-layer, or buckled layer, of NCs).

Fitting procedure for the XRR data

To describe the goodness of the fit, often χ^2 is used. But because the signal has a big part in the low intensity area, (50% of the signal is eight orders of magnitude less than the initial signal) the χ^2 test tends to ignore half of the signal. This is because the uncertainty in signal scales with the square root of its intensity. This means that the top of the signal will have an error 10000 times smaller than the lower intensity part. A logarithmic weight scaling test was implemented to account for the exponentially decreasing scattering intensity at higher scattering vectors. For this logarithmic scaling, a weight factor can be introduced which determines the how strong the weight scaling is dependent on the intensity of the measurement. This could cause a small discrepancy between the fit and the experimental data. Now if this discrepancy is larger than the Poissonian error box this completely destroys the effectiveness of the χ^2 test. Especially since this discrepancy would have an effect 1000 times bigger in the upper part of the curve than it would have in the lower part.

How do we know if adding a parameter adds statistical relevance to the model or the solution improves by coincidence? If a random feature is added to the system that does not exist, it might

just happen that the solutions improves, but this does not mean that we are closer to reality since the feature is completely unrelated to the physical system. This might also happen if we add a real parameter that we think is present but might not be. This is where the Fisher Criterion is implemented[2], which allows for calculations within 95% confidence if the added parameter really improves the solution with respect to reality.

Fishers Criterion states that the ratio of the residues should be higher than the value for which the Cumulative Fisher Distribution function reaches the predefined confidence interval to not be statistically identical.

Units throughout the XRR tables below are: Ångströms for all distances and sizes, such as NC diameters, surfactant shell sizes, interparticle distances, neck widths, etc. The angles are given in degrees. All ratios are given w.r.t. their main units, e.g. coverage = nanoparticle surface area (seen from the top) / total surface area upon close packing.

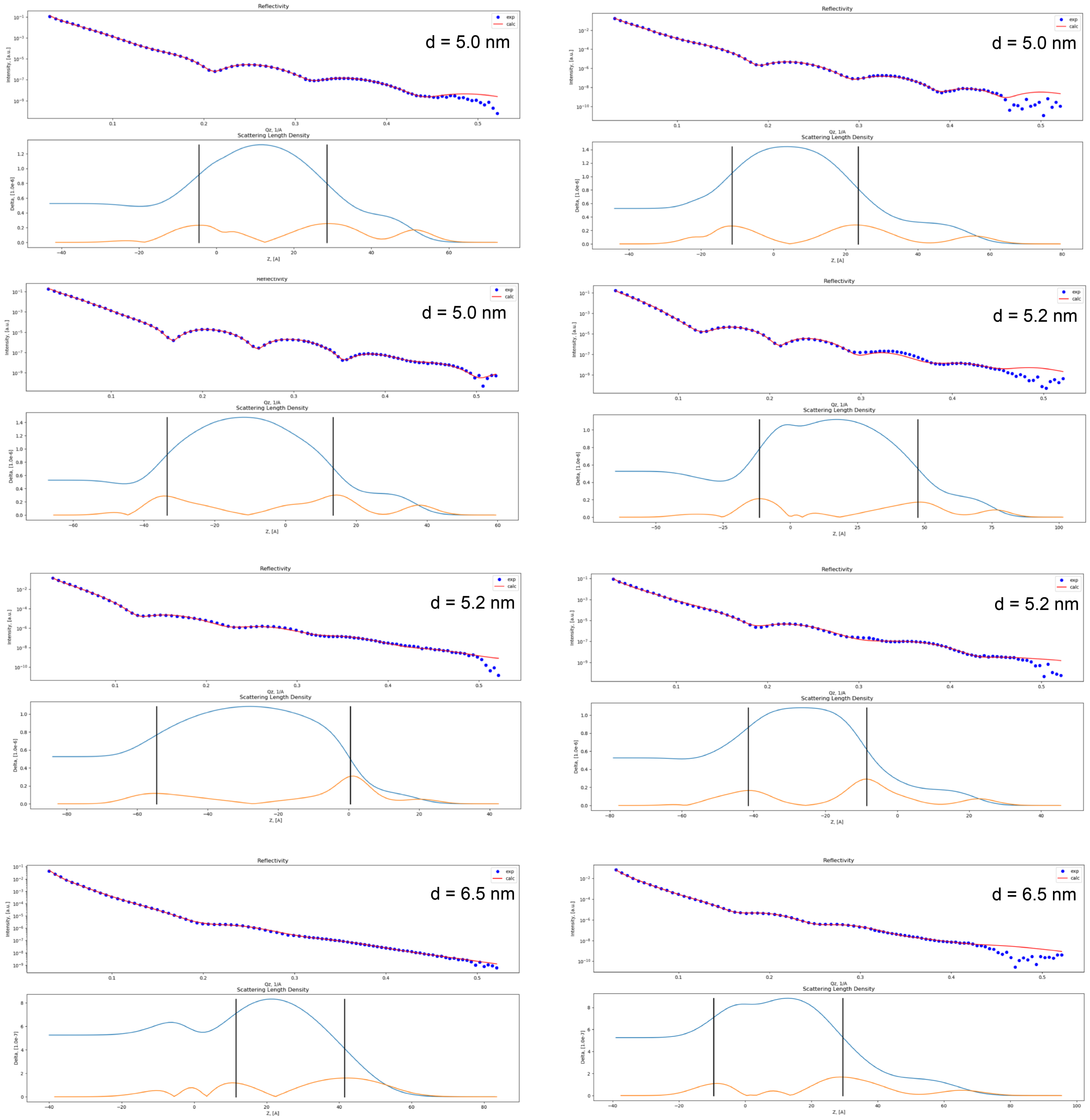

Supplementary Figure 14: XRR data with the single particle model fit, part 1. The above panels show the data and fit, the panels on the bottom show the density profile and first derivative of the density profiles.

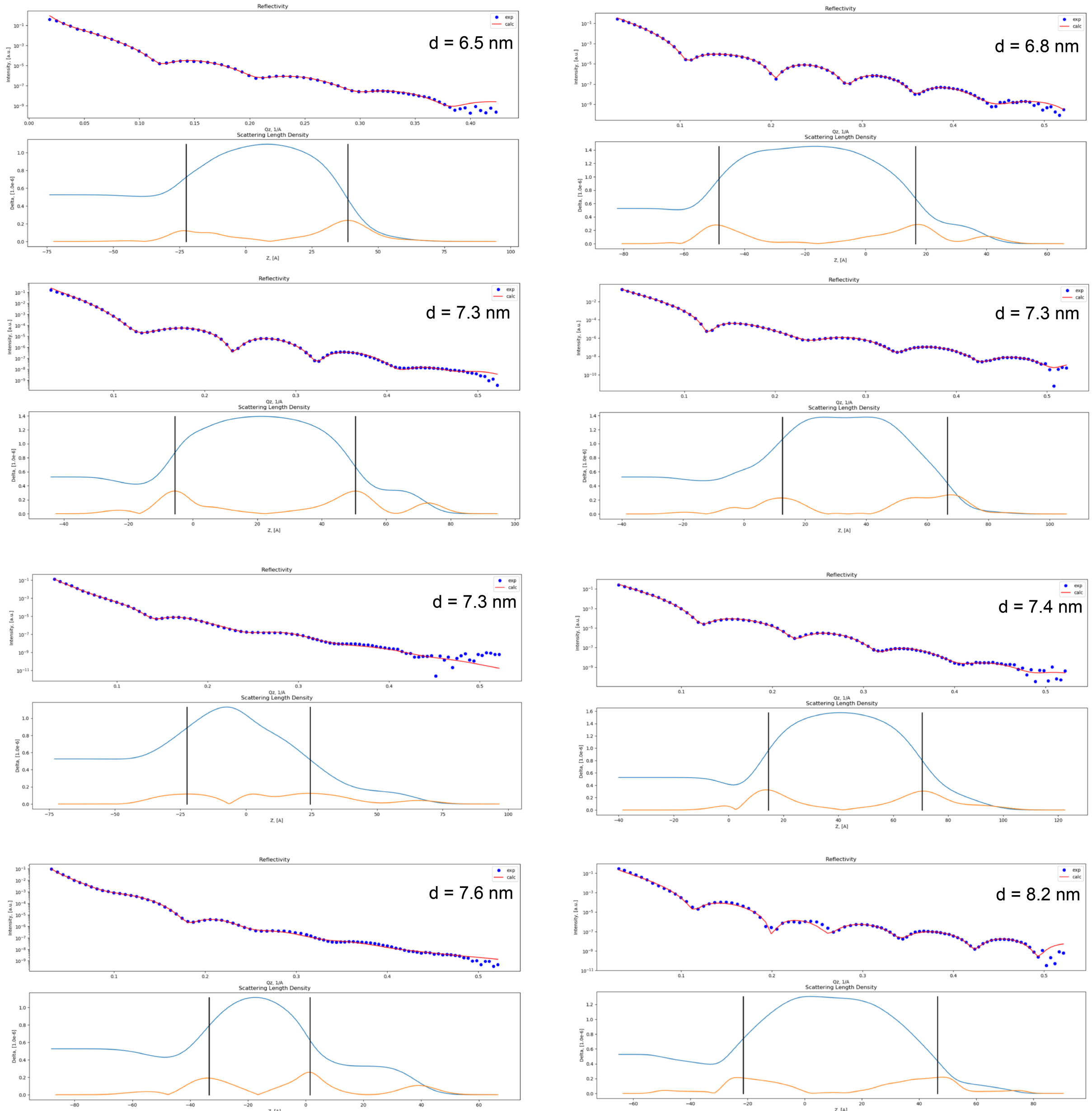

Supplementary Figure 15: XRR data with the single particle model fit, part 2. The above panels show the data and fit, the panels on the bottom show the density profile and first derivative of the density profiles.

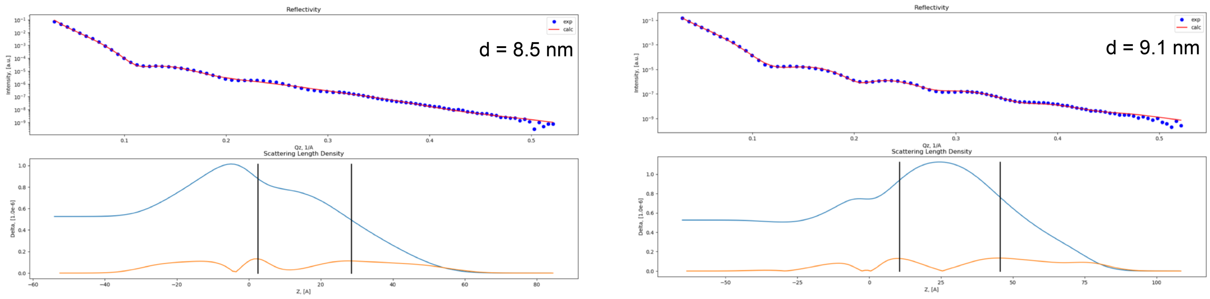

Supplementary Figure 16: XRR data with the single particle model fit, part 3. The above panels show the data and fit, the panels on the bottom show the density profile and first derivative of the density profiles.

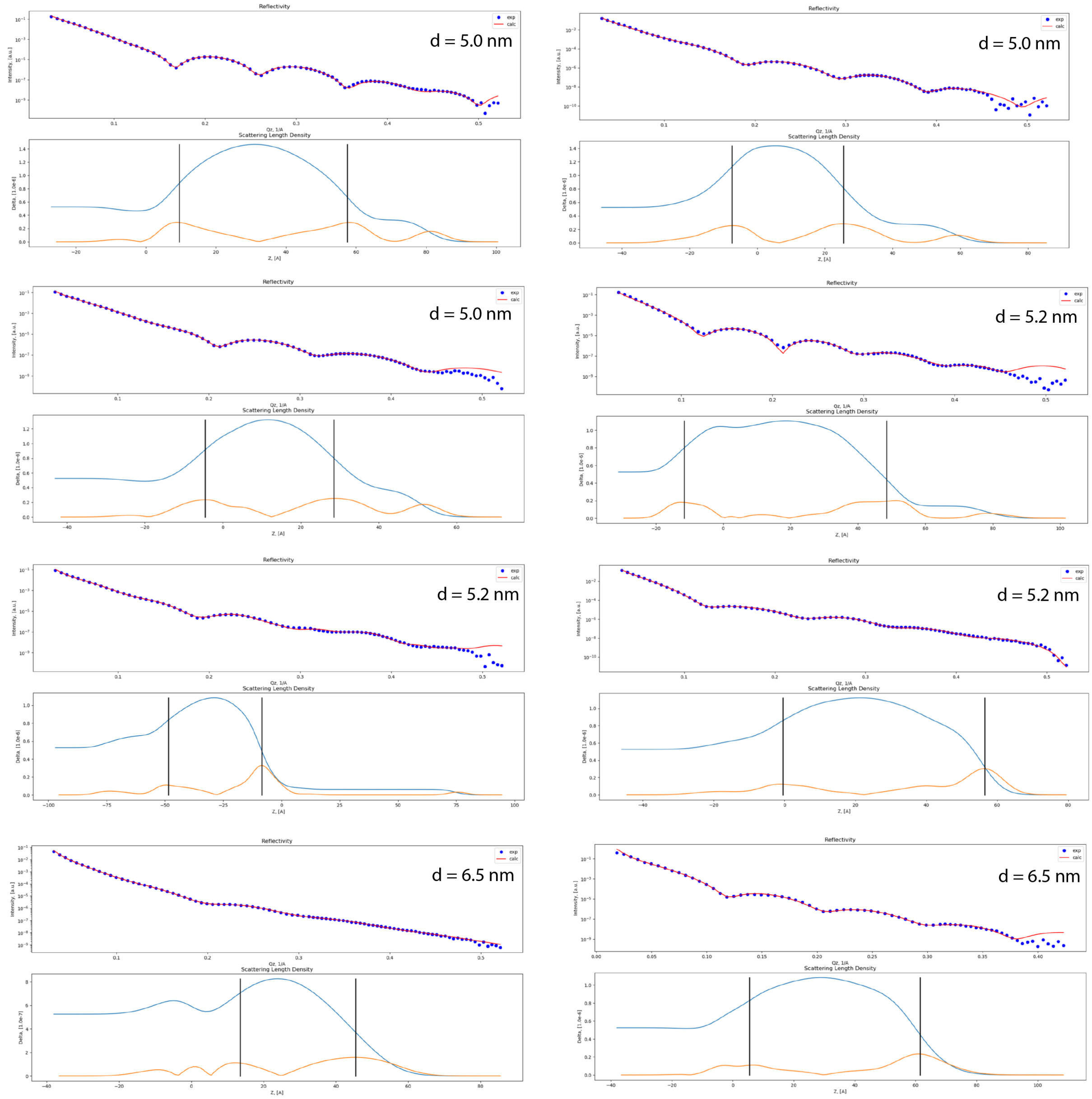

Supplementary Figure 17: XRR data with the double particle model fit, part 1. The above panels show the data and fit, the panels on the bottom show the density profile and first derivative of the density profiles.

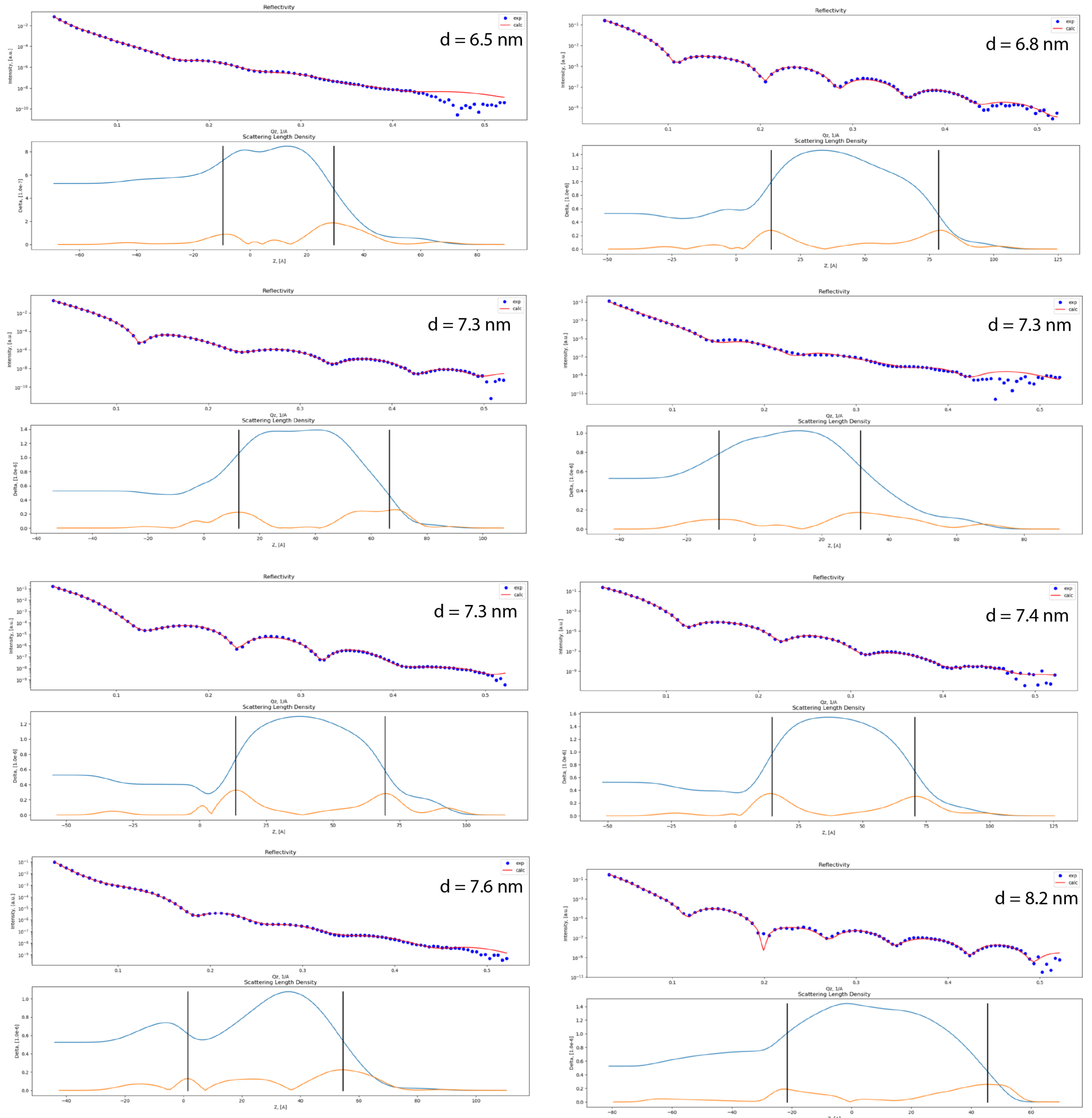

Supplementary Figure 18: XRR data with the double particle model fit, part 2. The above panels show the data and fit, the panels on the bottom show the density profile and first derivative of the density profiles.

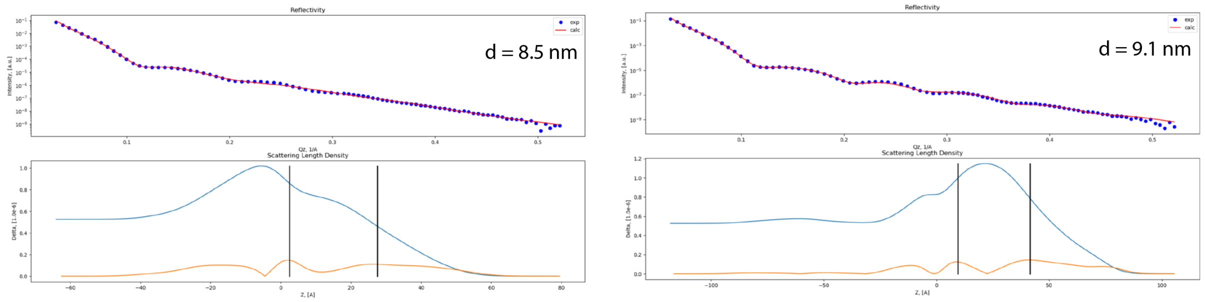

Supplementary Figure 19: XRR data with the double particle model fit, part 3. The above panels show the data and fit, the panels on the bottom show the density profile and first derivative of the density profiles.

Particle diameter TEM (nm)	6.5	8.5	9.1	6.8	5.0	7.3	7.6	5.2	6.5
Particle D	60.00	64.59	82.01	69.15	45.30	54.54	77.77	50.06	51.56
Shell Thickness	10.27	0.12	24.95	20.27	11.49	11.46	17.93	13.58	4.28
Particle Distance	82.95	87.10	85.30	87.65	60.91	65.00	73.51	62.99	62.99
Wetting Angle	136.07	102.38	108.66	146.14	109.65	177.84	128.12	45.27	172.58
Liquid Roughness	3.98	4.00	3.85	3.90	2.47	3.35	3.22	3.61	4.00
Particle Roughness	6.72	5.30	6.59	5.30	4.32	4.96	4.23	3.76	5.87
Bend Depth 1	-1.66	-3.86	-1.50	-19.18	-0.55	-3.23	-20.00	-16.38	-0.10
Bend/Unbend	0.08	0.68	0.85	0.58	0.15	0.64	0.33	1.00	0.00
Bend Depth 2	9.96	9.97	1.61	1.04	1.28	0.33	2.01	3.78	0.00
Surfactant Thickness	68.96	45.84	74.95	63.36	50.37	40.13	24.61	72.55	43.45
Surfactant Density	0.84	0.80	0.96	1.00	0.99	1.00	0.80	0.40	0.97
Shell Density	0.91	0.74	0.58	0.72	0.95	0.72	0.75	0.88	1.00
Truncation Ratio	0.10	0.63	0.78	0.35	0.59	0.72	0.61	0.86	0.67
Shell Density Air	0.99	0.73	0.75	0.92	0.99	0.61	0.61	0.72	0.81
Shell Density Liq	0.60	0.60	0.60	0.60	0.60	0.60	0.78	1.00	0.87
S Thickness ratio Air	0.99	1.64	0.50	0.99	0.86	1.87	0.94	0.93	0.80
S Thickness ratio Liq	0.87	1.97	1.25	1.95	1.15	0.84	1.07	0.67	0.97
S Thickness Truncated	0.43	0.40	0.16	0.99	1.02	0.00	1.15	1.03	1.05
Density Surfactant Air	1.04	0.75	0.31	1.00	1.16	0.70	1.00	0.92	0.68
Density Surfactant Liq	0.86	0.98	1.00	1.03	1.00	1.00	0.74	0.87	1.00
Coverage	0.58	0.46	0.61	0.62	0.84	0.66	0.60	0.40	0.40
Neck Width	15.15	0.62	9.76	19.93	7.14	6.23	13.68	19.99	15.31
Neck Occupation	1.00	0.45	0.42	0.97	0.94	0.99	0.74	1.00	0.74
Particle 2 Offset	0.92	15.70	70.61	25.57	0.90	33.74	32.14	27.95	17.16
Particle 2 Occupancy	0.10	0.26	0.06	0.55	0.01	0.36	0.00	0.42	0.74

Supplementary Table 5: Fit results for the double particle model, part 1.

Particle diameter TEM (nm)	6.5	7.4	8.2	7.3	5.2	5.0	7.3	5.0	5.2
Particle D	60.00	64.59	82.01	69.15	45.30	54.54	77.77	50.06	51.56
Shell Thickness	10.27	0.12	24.95	20.27	11.49	11.46	17.93	13.58	4.28
Particle Distance	82.95	87.10	85.30	87.65	60.91	65.00	73.51	62.99	62.99
Wetting Angle	136.07	102.38	108.66	146.14	109.65	177.84	128.12	45.27	172.58
Liquid Roughness	3.98	4.00	3.85	3.90	2.47	3.35	3.22	3.61	4.00
Particle Roughness	6.72	5.30	6.59	5.30	4.32	4.96	4.23	3.76	5.87
Bend Depth 1	-1.66	-3.86	-1.50	-19.18	-0.55	-3.23	-20.00	-16.38	-0.10
Bend/Unbend	0.08	0.68	0.85	0.58	0.15	0.64	0.33	1.00	0.00
Bend Depth 2	9.96	9.97	1.61	1.04	1.28	0.33	2.01	3.78	0.00
Surfactant Thickness	68.96	45.84	74.95	63.36	50.37	40.13	24.61	72.55	43.45
Surfactant Density	0.84	0.80	0.96	1.00	0.99	1.00	0.80	0.40	0.97
Shell Density	0.91	0.74	0.58	0.72	0.95	0.72	0.75	0.88	1.00
Truncation Ratio	0.10	0.63	0.78	0.35	0.59	0.72	0.61	0.86	0.67
Shell Density Air	0.99	0.73	0.75	0.92	0.99	0.61	0.61	0.72	0.81
Shell Density Liq	0.60	0.60	0.60	0.60	0.60	0.60	0.78	1.00	0.87
S Thickness ratio Air	0.99	1.64	0.50	0.99	0.86	1.87	0.94	0.93	0.80
S Thickness ratio Liq	0.87	1.97	1.25	1.95	1.15	0.84	1.07	0.67	0.97
S Thickness Truncated	0.43	0.40	0.16	0.99	1.02	0.00	1.15	1.03	1.05
Density Surfactant Air	1.04	0.75	0.31	1.00	1.16	0.70	1.00	0.92	0.68
Density Surfactant Liq	0.86	0.98	1.00	1.03	1.00	1.00	0.74	0.87	1.00
Coverage	0.58	0.46	0.61	0.62	0.84	0.66	0.60	0.40	0.40
Neck Width	15.15	0.62	9.76	19.93	7.14	6.23	13.68	19.99	15.31
Neck Occupation	1.00	0.45	0.42	0.97	0.94	0.99	0.74	1.00	0.74
Particle 2 Offset	0.92	15.70	70.61	25.57	0.90	33.74	32.14	27.95	17.16
Particle 2 Occupancy	0.10	0.26	0.06	0.55	0.01	0.36	0.00	0.42	0.74

Supplementary Table 6: Fit results for the double particle model, part 2.

Particle diameter TEM (nm)	6.5	8.5	9.1	6.8	5.0	7.3	7.6	5.2	6.5
Particle D	60.78	68.00	82.00	71.24	45.34	47.55	73.34	55.47	64.16
Shell Thickness	18.56	0.03	16.38	9.39	12.44	19.38	16.74	18.14	0.00
Particle Distance	82.62	84.01	87.99	86.51	60.61	64.07	76.00	62.56	63.00
Wetting Angle	100.26	100.15	114.48	68.21	109.03	66.12	128.53	54.61	131.18
Liquid Roughness	4.00	3.94	3.79	5.02	1.59	3.50	3.44	4.17	3.95
Particle Roughness	7.00	5.92	6.93	4.81	4.60	6.20	4.52	4.51	6.96
Bend Depth 1	-6.96	-0.52	-2.12	-5.77	-16.84	-4.28	-5.34	-0.50	-0.10
Bend/Unbend	0.51	0.46	0.45	0.47	0.34	0.83	0.95	0.97	0.00
Bend Depth 2	9.99	9.97	1.41	5.99	1.92	0.40	0.72	5.09	0.00
Surfactant Thickness	54.00	47.95	75.63	37.99	49.92	37.99	67.38	19.27	46.71
Surfactant Density	0.90	0.71	1.00	0.88	0.84	0.92	0.61	0.98	-1.34
Shell Density	0.02	0.95	0.99	0.95	0.53	1.00	0.86	1.00	3.36
Truncation Ratio	0.30	0.64	0.76	0.36	0.62	0.15	0.60	0.64	0.66
Shell Density Air	0.55	0.53	1.00	0.96	0.72	1.00	0.50	1.00	0.55
Shell Density Liq	1.00	0.60	0.50	0.60	0.60	0.60	0.60	0.50	0.76
S Thickness ratio Air	0.98	0.74	0.51	0.64	0.70	0.60	0.85	0.53	0.64
S Thickness ratio Liq	1.20	0.76	1.12	0.98	1.02	0.77	1.03	0.30	0.53
S Thickness Truncated	0.44	0.76	0.50	0.60	0.46	0.54	0.88	1.19	1.12
Density Surfactant Air	0.65	1.20	1.00	1.17	1.19	1.17	1.19	1.00	0.30
Density Surfactant Liq	1.00	0.88	0.50	0.95	0.63	0.84	0.88	0.79	1.20
Coverage	0.68	0.48	0.60	0.63	0.91	0.67	0.62	0.40	0.40
Neck Width	13.31	18.38	8.09	11.70	6.14	0.97	10.10	19.78	0.60
Neck Occupation	0.08	0.95	1.00	0.92	0.90	1.00	0.01	1.00	0.76

Supplementary Table 7: Fit results for the single particle model, part 1.

Particle diameter TEM (nm)	7.4	5.2	6.5	7.3	8.2	5.0	7.3	5.0	5.2
Particle D	74.59	54.97	70.52	59.45	80.94	62.75	72.00	50.72	52.71
Shell Thickness	23.98	0.12	0.00	17.54	25.00	20.39	13.94	1.40	17.36
Particle Distance	82.56	64.91	79.00	77.27	88.83	76.89	74.98	60.48	79.88
Wetting Angle	106.37	116.97	37.23	116.77	100.15	141.77	90.00	100.01	75.16
Liquid Roughness	3.38	3.82	4.03	3.00	6.00	3.92	5.57	2.36	4.15
Particle Roughness	4.82	5.38	4.86	4.59	2.53	6.31	7.00	5.31	4.59
Bend Depth 1	-1.55	-0.61	-2.23	-19.99	-2.28	-13.78	-4.78	-10.31	-5.80
Bend/Unbend	0.57	0.73	0.00	1.00	0.23	0.00	0.57	0.38	0.22
Bend Depth 2	6.00	10.00	0.96	2.48	0.63	8.97	1.18	1.43	2.60
Surfactant Thickness	74.00	61.00	19.10	70.68	36.63	85.37	64.71	54.08	36.01
Surfactant Density	0.75	0.50	0.57	0.96	0.64	1.00	0.83	0.93	0.78
Shell Density	1.00	0.61	0.78	0.73	0.58	0.82	1.00	0.01	0.76
Truncation Ratio	0.22	0.75	0.11	0.34	0.68	0.34	0.66	0.61	0.29
Shell Density Air	1.00	0.51	0.54	0.97	1.00	0.82	1.00	0.10	0.88
Shell Density Liq	0.60	0.60	0.60	0.60	0.60	0.60	0.60	1.10	0.60
S Thickness ratio Air	0.62	0.51	0.57	0.57	0.89	1.10	1.05	0.67	0.80
S Thickness ratio Liq	0.93	0.54	0.73	0.89	0.86	0.50	0.51	0.60	0.55
S Thickness Truncated	0.38	0.77	0.82	0.34	0.49	0.43	0.41	0.74	1.02
Density Surfactant Air	0.93	1.14	1.20	1.11	1.20	1.20	0.96	0.94	1.12
Density Surfactant Liq	0.83	0.50	0.58	0.50	0.77	0.59	0.73	1.03	0.51
Coverage	0.58	0.60	0.40	0.76	0.68	0.79	0.41	0.81	0.90
Neck Width	0.76	0.57	0.55	17.79	0.56	15.95	11.12	19.69	17.72
Neck Occupation	0.31	0.10	0.80	0.64	0.73	0.99	0.87	0.78	0.73

Supplementary Table 8: Fit results for the single particle model, part 2.

Particle Diameter (nm)	6.5	8.5	9.1	6.8	5.0	7.6	7.3	5.2	6.5
χ^2	0.0009	0.0064	0.0031	0.0028	0.0050	0.0007	0.0014	0.0051	0.0023
fishers criterion	0	0	0	1	0	1	0	0	0
Log-test	1.48	2.32	1.51	2.04	0.66	0.91	0.65	2.31	0.63
fishers criterion	0	0	0	0	0	1	0	1	0

Particle Diameter (nm)	6.5	5.2	7.3	8.2	7.4	7.3	5.0	5.0	5.2
χ^2	0.0003	0.0017	0.0001	0.0056	0.0025	0.0083	0.0034	0.0052	0.0081
Fishers criterion	0	0	1	1	1	1	0	1	0
Log-test	0.89	1.43	1.45	5.91	0.89	8.38	0.88	1.45	5.11
Fishers criterion	0	1	0	1	0	0	0	0	0

Supplementary Table 9: Table that shows the Goodness-of-Fit for the double particle model.

Numerical method for calculating the adsorption geometry of the truncated PbSe nanocrystals.

Here we provide a more extensive illustration of the numerical method of Soligno *et al.*[3–5], used for the interface-adsorption calculations presented in this paper. We consider a macroscopic model where the interface between two homogeneous fluids, fluid 1 (air) and fluid 2 [toluene or ethylene glycol (EG)], is treated as a 2D possibly-curved surface, which we represent by a 2D triangular grid of points. A PbSe nanocrystal (NC) adsorbed at this interface is modeled as a homogeneous solid with surface represented by a 2D closed triangular grid of points. We consider two possible shapes for the NC, a rhombicuboctahedron and a cantellated rhombicuboctahedron. For the cantellated rhombicuboctahedron, the degree of cantellation is such that the side of a {100} facet is 0.75 times the distance between two opposite {100} facets.

We numerically calculate the energy

$$U = \gamma A + \gamma_1 A_1 + \gamma_2 A_2 \qquad \text{(Supplementary equation 4)}$$

of the NC-fluid-fluid system with respect to the NC position and orientation at the interface, where A_1, A_2 is the surface area of the particle in contact with fluid 1 and fluid 2, respectively, γ_1, γ_2 is the surface tension of the particle surface with fluid 1 and fluid 2, respectively, A is the fluid-fluid surface area, and γ is the fluid-fluid surface tension. Note that in general the actual thermodynamic potential minimized at equilibrium by a NC at the fluid-fluid interface (assuming constant the temperature, the total volume of the system, and the chemical potentials of the two fluids) is[5]

$$\Omega = \gamma A + \gamma_1 A_1 + \gamma_2 A_2 - P_1 V_1 - P_2 V_2 \qquad \text{(Supplementary equation 5)}$$

where P_1, P_2 is the pressure of fluid 1 and fluid 2, respectively, and V_1, V_2 is the volume of fluid 1 and fluid 2, respectively. Gravity is not taken into account in Eq. (S2) since negligible for nanoparticles. The terms $- P_1V_1 - P_2V_2$ can be rewritten as $-(P_2- P_1)V_2- P_1(V_1+V_2)$. The term $P_1(V_1+V_2)$ is constant, since the total volume of the system is constant, and hence it is negligible. The pressure difference $P_2- P_1$ is zero, since we assume a flat fluid-fluid interface far away from the NC. So, the terms $- P_1V_1 - P_2V_2$ can be neglected in Supplementary equation 5, that is U is equivalent to Ω.

In Soligno's numerical method,[4,5] the fluid-fluid interface is represented by a 2D triangular grid of points, and a simulated annealing algorithm is used to find the point position that minimizes U, that is the equilibrium shape of the fluid-fluid interface.[34] Given the desired position and orientation of the NC at the fluid-fluid interface, first the equilibrium shape of the fluid-fluid interface is computed, and then U is obtained by numerically calculating A, A_1, A_2 from the equilibrium shape of the fluid-fluid interface. By repeating this procedure for different orientations of the NC, the U landscape with respect to the NC orientation at the interface is obtained. In the calculations presented in this paper, the equilibrium shape of the fluid-fluid interface is computed allowing exchange of volume between the two fluids. Therefore, the obtained equilibrium shape of the fluid-fluid interface will have minimum energy U with respect to the height of the NC center of mass on the fluid-fluid interface level far away from the NC. To simulate a flat fluid-fluid interface far away from the NC, we use in our model a vertical wall with Young's contact angle $\pi/2$ to enclose the NC-fluid-fluid system, and we place this wall far enough from the NC to avoid finite-size effects. The initial shape of fluid-fluid interface grid before starting a simulated annealing simulation to minimize E is the plane z=0.

For convenience, we shift U by a constant and define

$$E \equiv U - \gamma A_0 - \gamma_2(A_1 + A_2) \qquad \text{(Supplementary equation 6)}$$

where both A_0 (the area of the fluid-fluid interface when no NC is present) and A_1+A_2 are constants. Using Eq. Supplementary Equation 3, E can be written as

$$E = \gamma(A - A_0 + A_1 \cos\theta) \qquad \text{(Supplementary equation 7)}$$

where Young's Law [34]

$$\cos\theta = (\gamma_1 - \gamma_2)/\gamma \qquad \text{(Supplementary equation 8)}$$

has been used. Note that E=0 when the NC is desorbed from the interface and fully immersed in fluid 2, i.e. when A=A_0 and A_1=0.

To represent a NC capped by oleic acid at a toluene/air interface, we assume that the surface tension γ_1, γ_2 of the NC surface with air and toluene is approximately similar to the surface tension of hexane with air (0.018 N/m at room temperature)[6] and toluene (0 at room temperature, since hexane is miscible in toluene), respectively. So, given the toluene/air surface tension at room temperature γ=0.028 N/m,[6] one obtains $\cos\theta = (\gamma_1 - \gamma_2)/\gamma \approx 0.64$.

Analogously, to represent a NC capped by oleic acid at an ethylene glycol/air interface, we assume that the surface tension γ_1, γ_2 of the NC surface with air and ethylene glycol is approximately similar to the surface tension of hexane with air (0.018 N/m at room temperature)[6] and ethylene glycol (0.016 N/m at room temperature),[7] respectively. So, given the ethylene glycol/air surface tension at room temperature γ=0.047 N/m,[6] one obtains $\cos\theta = (\gamma_1 - \gamma_2)/\gamma \approx 0.05$.

Finally, to represent a NC capped by oleic acid at an ethylene glycol/toluene interface, we assume that the surface tension γ_1, γ_2 of the NC surface with ethylene glycol and toluene is approximately similar to the surface tension of hexane with ethylene glycol (0.016 N/m at room temperature),[7] and toluene (0 at room temperature, since hexane is miscible in toluene), respectively. At the best of the authors' knowledge, the surface tension between ethylene glycol and toluene at room temperature is $\gamma \approx 0.01$ N/m,[8] implying that $\cos\theta = (\gamma_1 - \gamma_2)/\gamma > 1$. This means that a NC capped by oleic acid does not adsorb at an ethylene glycol/toluene interface and prefers instead to stay in the toluene phase (i.e. without touching the ethylene glycol).

We would like to point out that the surface tensions we use are reasonable estimates, since, to our knowledge, there is no better experimental data or theoretical prediction available. We reasonably expect that the surface tension of the oleate-capped NC's surface with the surrounding medium is similar to the surface tension of hexane with the same medium.

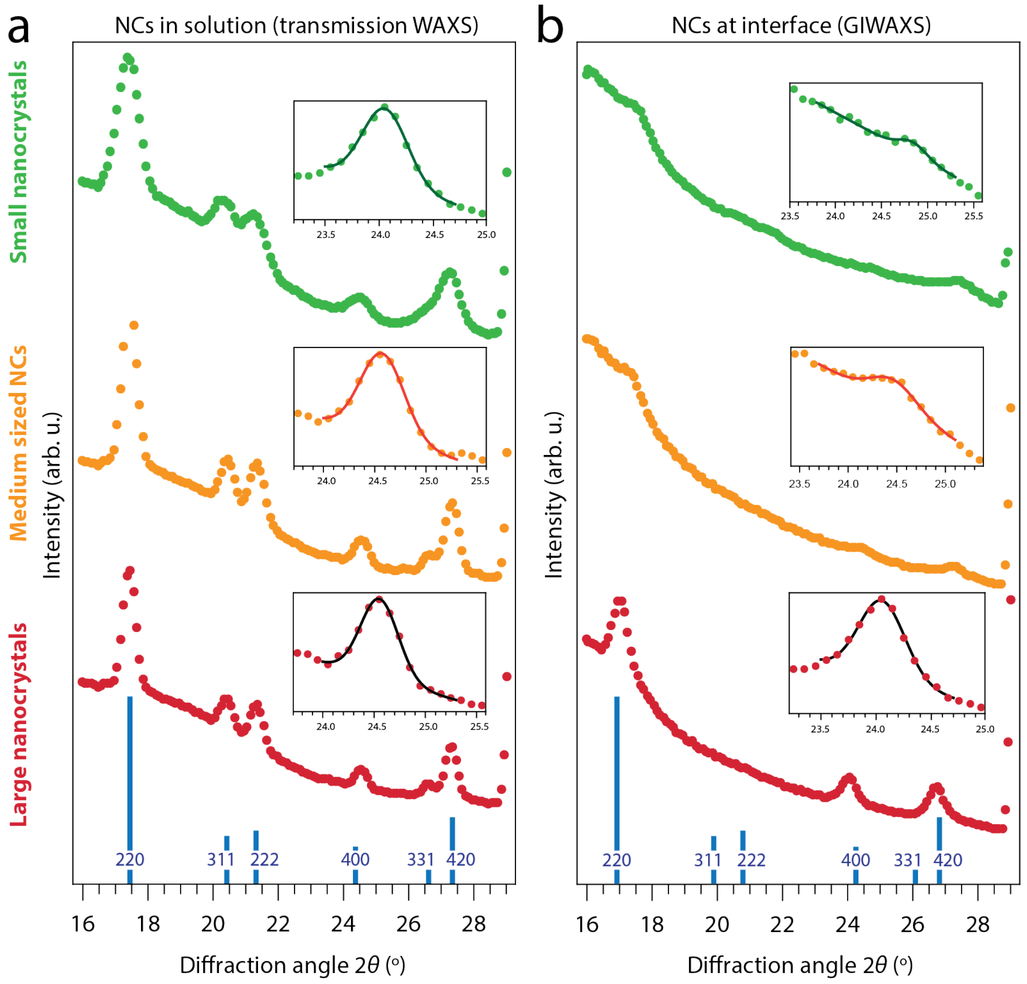

Supplementary Figure 20: Intensity traces in along the 2*θ* direction of the WAXS signal. The traces were obtained by integrating the signal in the direction of the azimuthal angle from 1-3° to increase the signal-to-noise ratio. (a) Transmission WAXS signal of the small (green), medium-sized (yellow) and large (red) NC solutions as presented throughout the main text. The insets show the WAXS signal around the reflection originating from the {400} lattice planes and a Gaussian fit to obtain the FWHM. (b) GIWAXS signal of the NC monolayer, obtained by integrating the signal displayed in Figure 3 of the main text in the azimuthal angle direction from 1-3°. Note how for the medium-sized and large NCs only reflections originating from {*hk*0} lattice planes are visible, which indicates that the NCs have a {001} facet pointing upwards.

	Solution FWHM (deg)	Solution diameter (nm)	Interface FWHM (deg)	Interface diameter (nm)
Small NCs	0.57	5.8	0.59	5.6
Medium sized NCs	0.47	7.0	0.45	7.4
Large NCs	0.40	8.3	0.38	8.8

Supplementary Table 10: Acquired FWHM from the data in Supplementary Figure 17 of the 400 reflection and the corresponding crystalline NC size. The acquired crystalline sizes of the NCs match the NC diameters obtained with TEM (5.2 nm, 7.3 nm and 8.2 nm respectively) quite well. No significant increase of crystalline size is observed compared to the NC diameter, indicating that the NCs did not fuse together atomically in the performed experiments.

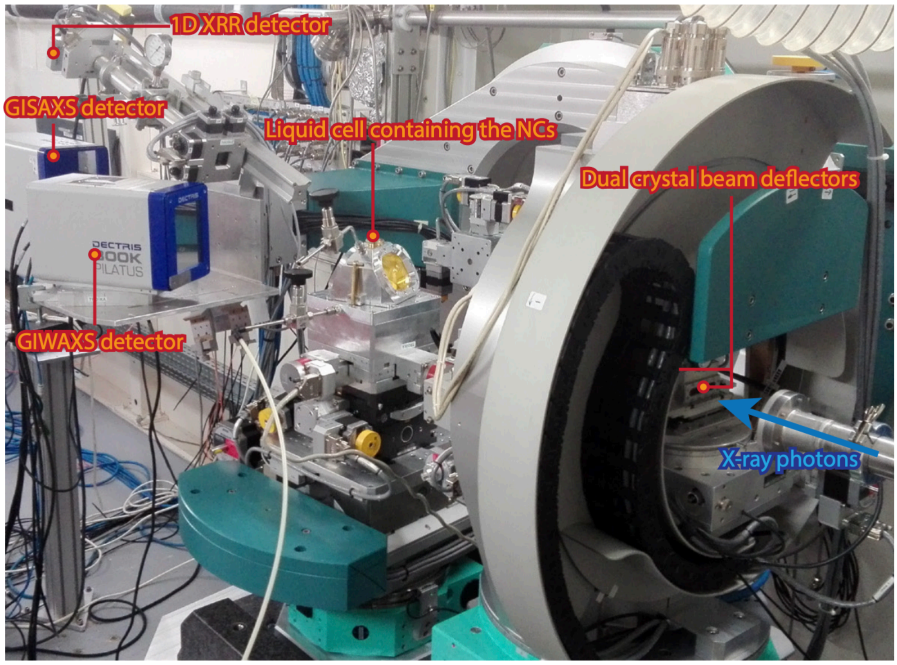

Supplementary Figure 21: Photograph of the experimental geometry at ID10 beamline, ESRF. The X-ray beam is set to have an energy of 22 keV. The GISAXS signal is collected in the forward scattering direction at a sample-to-detector distance of 988 mm. The GIWAXS detector is placed closer to the liquid cell and at a higher angle to capture the atomic diffraction. After complete solvent evaporation, the arm of the diffractometer is lowered to perform XRR experiments. To vary the angle of incidence, we use the new diffractometer of ID10 with a double crystal deflector to bend the beam down. The intensity of the specular reflection is collected on the 1D XRR detector.

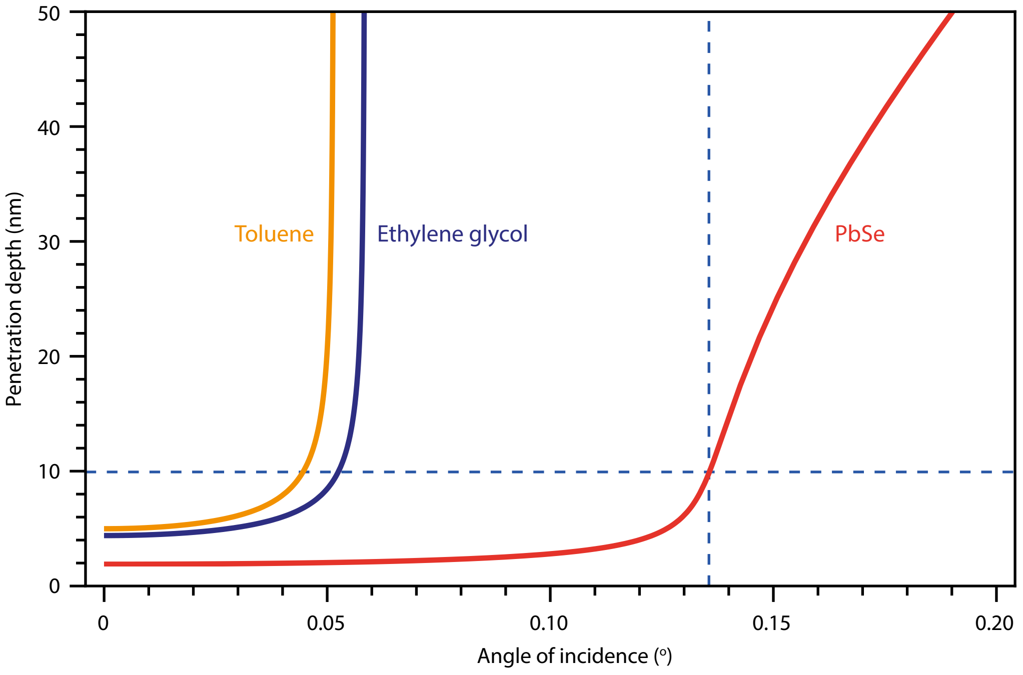

Supplementary Figure 22: Calculation of the X-ray penetration depth as a function of inc ident angle. The red, blue and yellow lines depict the penetration depths as function of inciden t angle for PbSe, ethylene glycol and toluene respectively. The blue dashed lines show the incident angle of the experiments (0.14°) and the corresponding penetration depth for PbSe (10 nm). Working at the critical angle of either toluene or ethylene glycol would result in a minute penetration of the X-ray photons into the PbSe superstructure.

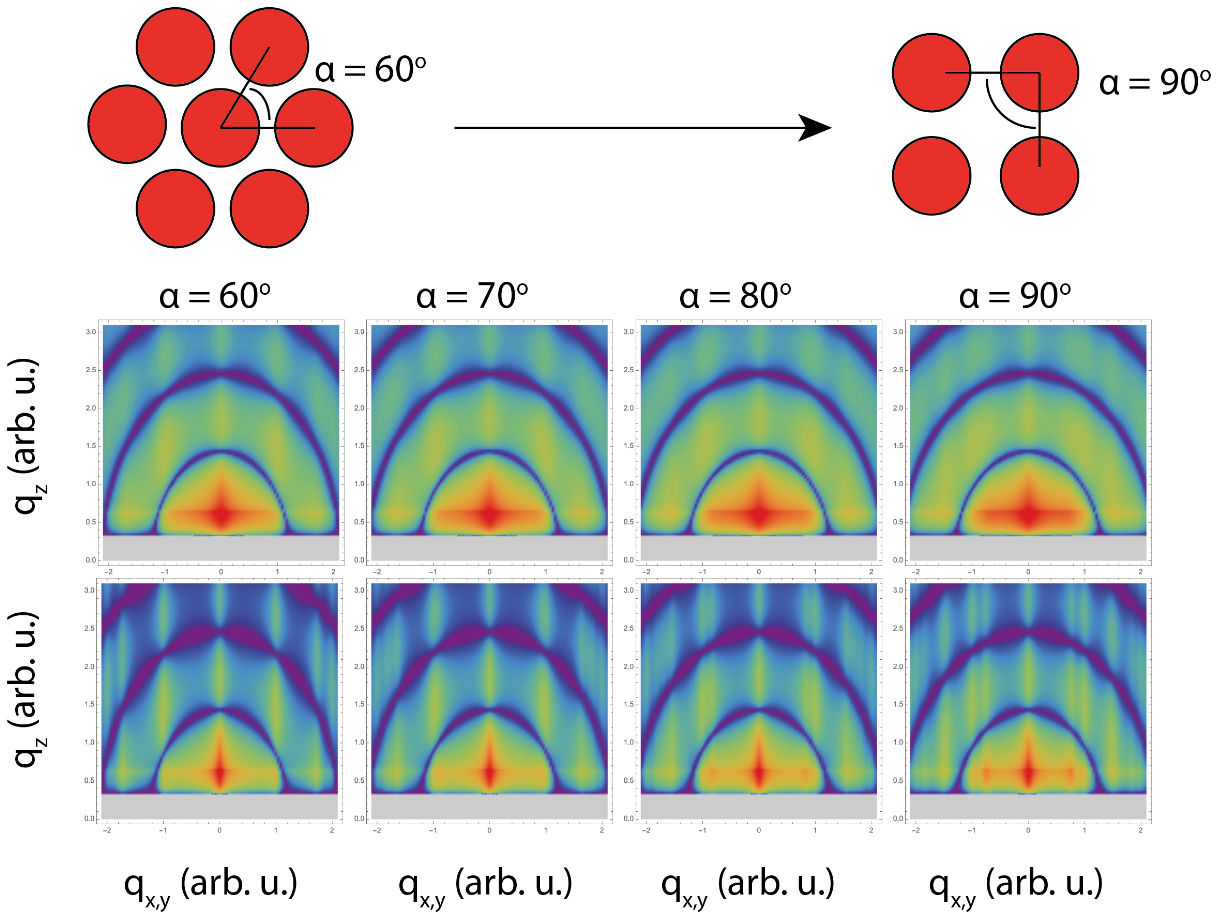

Supplementary Figure 23: Simulations of disorder on the GISAXS pattern of 2D self-assembled nanocrystal lattices. We varied (1) the angle between the nanocrystals, going from an hexagonal to square two-dimensional structure, and (2) the amount of disorder (related to the domain size) in the superlattice, by adjusting the broadening of the structure factor peaks. In the bottom row, we use a relatively small broadening (1%) whereas in the top row, we increased the amount of broadening (to about 6%). Note that **we did not adjust disorder in particle sizes**, since this would decrease the depth of the minima in the form factor.

SUPPLEMENTARY REFERENCES

(1) Steckel, J. S.; Yen, B. K. H.; Oertel, D. C.; Bawendi, M. G. On the Mechanism of Lead Chalcogenide Nanocrystal Formation. *J. Am. Chem. Soc.* **2006**, *128*, 13032–13033.

(2) Snedecor, G. W. (George W.; Cochran, W. G. (William G. *Statistical Methods*; Iowa State University Press, 1989.

(3) Soligno, G.; Dijkstra, M.; van Roij, R. Self-Assembly of Cubes into 2D Hexagonal and Honeycomb Lattices by Hexapolar Capillary Interactions. *Phys. Rev. Lett.* **2016**, *116*, 258001.

(4) Soligno, G.; Dijkstra, M.; van Roij, R. Self-Assembly of Cubic Colloidal Particles at Fluid–Fluid Interfaces by Hexapolar Capillary Interactions. *Soft Matter* **2018**, *14*, 42–60.

(5) Soligno, G.; Dijkstra, M.; van Roij, R. The Equilibrium Shape of Fluid-Fluid Interfaces: Derivation and a New Numerical Method for Young's and Young-Laplace Equations. *J. Chem. Phys.* **2014**, *141*, 244702.

(6) Smallwood, I. M. *Handbook of Organic Solvent Properties*; Arnold, 1996.

(7) Zografi, G.; Yalkowsky, S. H. Interfacial Properties of Polar Liquids Against Nonpolar Phases. *J. Pharm. Sci.* **1974**, *63*, 1533–1536.

(8) Aveyard, R.; Binks, B. P.; Fletcher, P. D. I.; Kirk, A. J.; Swansbury, P. Adsorption and Aggregation of Sodium Bis(2-Ethylhexyl) Sulfosuccinate in Systems Containing Toluene plus Water, 1,2-Ethanediol or 1,2,3-Propanetriol. *Langmuir* **1993**, *9*, 523–530.
